# C-reactive protein in degenerative aortic valve stenosis

**DOI:** 10.1186/1476-7120-4-24

**Published:** 2006-06-14

**Authors:** Pedro L Sanchez, AnnaMaria Mazzone

**Affiliations:** 1 Instituto de Ciencias del Corazón (ICICOR), Hospital Clínico Universitario de Valladolid, C/Ramón y Cajal n° 3, 47005, Valladolid, Spain; 2 Department of Cardiology and Cardiac Surgery, CNR Institute ofClinical Physiology, Ospedale Pasquinucci, Massa, Italy

## Abstract

Degenerative aortic valve stenosis includes a range of disorder severity from mild leaflet thickening without valve obstruction, "aortic sclerosis", to severe calcified aortic stenosis. It is a slowly progressive active process of valve modification similar to atherosclerosis for cardiovascular risk factors, lipoprotein deposition, chronic inflammation, and calcification. Systemic signs of inflammation, as wall and serum C-reactive protein, similar to those found in atherosclerosis, are present in patients with degenerative aortic valve stenosis and may be expression of a common disease, useful in monitoring of stenosis progression.

## Introduction

Degenerative aortic stenosis (AS) is at present time the most frequent valvulopaty in developed countries, and as life expectancy increases, the incidence and prevalence of AS will also rise fundamentally at the expense of the degenerative form.

The scientific interest of this valvar disorder was parked for many years, as the image tools necessary to quantify it are at our disposal and we have a certain agreement of the clinical moment for indicating the valve replacement. Nevertheless, in the past years different studies have demonstrated a common pathogenic mechanism between degenerative AS and atherosclerosis [1-3]. This is consistent with histopathological evidence that the lesions in AS involve active cellular processes that have classical "response to injury features", namely inflammatory infiltrates containing macrophages, T cells, and smooth muscle cells [4]. Therefore, investigators have needed only a half turn of the head to transfer the early past experience of blood markers of inflammation in the atherosclerosis setting to the AS scenario.

C-reactive protein (CRP) is the marker of inflammation most widely studied in patients with coronary artery disease and hence has become the marker of reference for any other inflammatory-based disease [5]. On this basis, CRP has emerged as a leading candidate for a better understanding of AS pathogenesis, for predicting AS progression, and for driving therapies in AS.

## C-reactive protein is increased in patients with degenerative aortic stenosis

The first study showing increased CRP levels in patients with degenerative AS was published by Galante et al, in 2001 [6]. They compared serum CRP levels of 68 consecutive patients with severe degenerative trileaflet AS and absence of coronary atherosclerotic lesions admitted for elective cardiac surgery with 92 healthy controls. CRP levels were higher in patients with AS than in controls (0.85 ± 1.42 vs. 0.39 ± 0.50; p = 0.0001). They also showed an independent association of CRP with AS; the odds ratio for the disease according to CRP levels was 2.62 (95% confidence interval: 1.06 to 6.49). No association between CRP and aortic jet velocity, aortic valve area, or degree of calcification was found, notwithstanding that all patients had severe aortic stenosis and were waiting for surgery.

Further studies have confirmed [1] and expanded these observations. Serum CRP is also elevated in patients with asymptomatic aortic stenosis [7,8], does not rise in accordance with increasing severity of valvar disease [7,8], and decreased from before to 6 months after aortic valve replacement [9]. However, other studies have shown a week association of CRP and AS in the initial stages of the disease (aortic sclerosis) [10].

One of the principal characteristics of AS is the important degree of valvar calcification that takes place along the different stages of the disease. Thus, investigating whether CRP could be related to the mechanism of calcification appears very atractive. The role of serum CRP in the tissue calcification process has been investigated in a recently elegant study by Warrier et al,. When aortic wall was exposed to an excess amount of CRP in an in vitro simulating model, the calcification rate of aortic wall increased as the concentration of CRP. The results of this work could reveal the role of CRP present in physiological fluid in aortic valvar calcification [11]. Data providing contribution of serum CRP to valve calcification in the clinical setting is available in patients with renal failure. Valvar calcification in patients with renal failure is associated with enhanced inflammation [12]. Furthermore, in chronic hemodyalisis patients in steady clinical conditions with no clinical evidence of either infectious or inflammatory diseases, a high CaxPO4 is associated with high CRP concentrations and hence associated with valvar calcification [13,14]. However, preliminary transversal evidence evaluating the association of CRP and calcification in patients with AS and no renal failure is controversial [6-8]; thus, the long-term predictive value of the serum CRP level for the development of aortic calcification should be addressed in future well-designed prospective trials.

Recently, Skowasch et al, have observed localization of CRP in valve tissue of degenerative AS and degenerative aortic valve bioprostheses [15]. Furthermore, serum CRP showed a significant correlation with the valvar CRP expression (r = 0.54; P < 0.001). Consequently, we must integrate our previous understanding of the physiological role of CRP in inflamed tissues, thereby promoting local anti-inflammatory and proinflammatory effects [16]. Therefore, elevated CRP levels, whether preceding the development of degenerative valvular aortic stenosis or established at some time during the course of the disease, may have a detrimental influence on the natural history of the disease by inducing local activation of complement and subsequent amplification of local inflammation and cellular damage [6,16-18].

## C-reactive protein and progression of aortic stenosis

We have assessed whether serum CRP levels could predict rapid AS progression. We measured serum high sensitivity CRP in 43 asymptomatic subjects with AS at baseline and six months later. Plasma CRP concentration was significantly higher in patients with rapid AS progression (5.1 [2.3 to 11.3] mg/L) compared to patients with slow AS progression (2.1 [1.0 to 3.1] mg/L, p = 0.007). The rate of progression was higher in patients who had a cutpoint level above the median CRP concentration (3 mg/L) than those who had levels ≤3 mg/L (66.7% vs 33.3%, p = 0.012 for aortic jet velocity and 62.5% vs 37.5%, p = 0.063 for aortic valve area. Little is known of the mechanisms responsible for progression of AS albeit mechanical, clinical, and metabolic variables have been suggested to contribute to rapid progression of AS [19,20]. Our data suggest that elevated CRP levels may be a marker of AS progression and could have important clinical implications as interventions that reduce CRP levels may be beneficial in the prevention of AS and perhaps also in reducing AS progression.

Finally, baseline CRP concentration was similar in patients who developed symptoms compared to those asymptomatic during follow-up. Data in patients with aortic sclerosis suggest a positive association between the risk of adverse cardiovascular events and the presence of coronary artery disease (hazard ratio [HR] 3.23, p = 0.003) and enhanced inflammation (HR 2.2, p = 0.001), and not as a result of the effects of valvar heart disease per se [21].

## Targeting C-reactive protein for the therapy of aortic stenosis

The presence of higher serum CRP levels and the tissue location of CRP in patients with AS have raised the important question of whether medical therapies with agents such as statins and ACE inhibitors, which have already been shown to delay the progression of atherosclerosis may also affect the progression of AS.

Preclinical studies have shown that experimental hypercholesterolemia provide evidence of a proliferative atherosclerosis-like process in the aortic valve that is inhibited by statins [22-24]; ie, atorvastatin inhibited calcification in the aortic valve by increasing eNOS protein and serum nitrite concentrations [23], and decreasing Lrp5 (low-density receptor-related protein) receptors involve in cellular proliferation and osteoblastogenesis via the beta-catenin signaling pathway [24].

Although recent retrospective clinical studies suggest that statins also may slow the hemodynamic progression of AS [25-29], the results of the SALTIRE study have been discouraging for all of us who believe that AS is an active disease process akin to atherosclerosis with lipoprotein deposition, chronic inflammation, and active leaflet calcification [30]. Therefore, it is important to analyze the neutral effect of high doses of atorvastatin in such an attractive hypothesis. First, the SALTIRE study differs not only because of its prospective design but also because the indications for therapy were different. In the retrospective trials, statin therapy was indicated for the treatment of hyperlipidemia, whereas in the prospective trial, patients in whom statins were indicated for the treatment of hyperlipidemia were excluded. Second, statin doses in the retrospective studies were lower. Finally, although the observation periods in the various studies were similar, patients in the retrospective studies were already receiving therapy at the time of inclusion in the study [31]. Aside from the already commented differences with retrospective studies, high proportion of patients were on drugs with anti-inflammatory effects (aspirin, betablockers or angiotensin converting enzyme inhibitors) that could have mitigated the pleiotropic effect of statins. For example, in the retrospective trials that found a lower rate of progression among patients treated with statins, the percentage of patients on aspirin was always important and significantly higher in the group with positive results [26,28,29]. Importantly, in the SALTIRE study, half of the patients in each group were on aspirin, a prevalence that would have a bearing on the results of the study. In addition, markers of inflammation would have been useful as a means to predict or monitor the individual's response to atorvastatin. If the authors were not just looking for a reduction cholesterol effects it is surprising that they have not yet provided any information about inflammatory effects. As the main finding, Skowasch et al, [15] in their recent study showed that both valvar CRP expression and serum CRP levels were found to be lower in patients on statins.

## Limitations

The main limitations of all the studies that have evaluated the association between CRP and AS are similar to the previous experience in the atherosclerosis setting: variability and laboratory methodology in CRP determination [32,33]; cardiovascular risk factors and other variables known to affect CRP concentrations [34]; CRP was determined once, so longitudinal data are not available; other non-inflammatory mechanisms are likely to have an important role in the pathogenesis of degenerative aortic valve disease [19,20]; small study populations; short follow-up intervals; etcetera.

## Conclusion

Moving forward, we must learn more about the pathogenic mechanisms of AS. We must integrate the atherosclerotic background of inflammatory biomarkers in our future research, and finally we must focus on the development of prospective, randomised trials using CRP to monitor the individual's response to treatments.

## Abbreviations

Angiotensin Converting ezyme (ACE)

Aortic stenosis (AS)

C-reactive protein (CRP)

Hazard Ration (HR)

## Competing interests

The author(s) declare that they have no competing interests.

## Authors' contributions

Both authors contributed equally to the review.
